# Access to Primary and Specialty Health Care in the California State Prison Population During the COVID-19 Pandemic

**DOI:** 10.1001/jamahealthforum.2022.1868

**Published:** 2022-07-15

**Authors:** Anita Amin, Daniel Winetsky, William L. Schpero

**Affiliations:** 1 Department of Population Health Sciences, Weill Cornell Medical College, New York, New York; 2 Division of Infectious Diseases, SUNY Downstate Health Sciences University, New York, New York

## Abstract

This cross-sectional study examines trends in referrals for and timely delivery of primary and specialty health care among individuals incarcerated in California state prisons during the COVID-19 pandemic.

## Introduction

The COVID-19 pandemic has diverted health care resources from the management of chronic disease toward acute care, with potential long-term consequences, especially for disadvantaged subgroups.^1^ Incarcerated populations have a higher chronic disease burden than individuals residing in the community and have experienced COVID-19 outbreaks with higher acuity and associated mortality.^2,3^ The extent to which COVID-19 disrupted access to medical care within prison health systems has not been fully characterized. We assessed trends in referrals for and timely delivery of primary and specialty health care among individuals incarcerated in California state prisons during the COVID-19 pandemic.

## Methods

This cross-sectional study analyzed data from the California Department of Corrections and Rehabilitation (CDCR) for all 35 state prisons to describe demographics of the prison population and referrals for and timely delivery of primary and specialty care by month from January 1, 2019, through July 31, 2021 (details are given in the eMethods in the Supplement).^4^ We plotted unadjusted trends in per capita referrals for primary and specialty care and the proportion of care delivered in a timely manner. Controlling for variation in prison-level age distribution, chronic disease prevalence, and disability prevalence over time, we calculated changes in care delivery during the early COVID-19 period (March to May 2020), first wave (June to October 2020), and second wave (November 2020 to February 2021) compared with corresponding prepandemic baseline periods in 2019 and 2020. Analyses were conducted using Stata/MP, version 16.1. Two-sided *P* < .05 was considered significant. This study followed the STROBE reporting guideline and was exempted from review by the institutional review board at Weill Cornell Medical College because it was deemed not human participants research.

## Results

A mean (SD) of 110 826 (11 513) individuals were incarcerated in California’s 35 state prisons during the study period. Although occupancy rates decreased from 2019 to 2021, individuals who remained incarcerated were older and had a higher burden of chronic disease (Table). Populations residing in CDCR facilities experienced an initial wave of COVID-19 cases from June to October 2020 (peak 14-day incidence, 17 cases per 1000 incarcerated persons), followed by a larger wave from November 2020 to February 2021 (peak 14-day incidence, 88 cases per 1000 incarcerated persons) (Figure).

**Table.** Characteristics of 35 California State Prisons and Prison Populations From January 2019 to July 2021

Characteristic	Mean (SD)^a^		
	2019	2020	2021^b^
Prison			
Institutional population, No.	121 437.8 (301.3)	108 544.4 (10 051.3)	96 543.4 (1915.2)
Occupancy, %^c^	131.1 (0.3)	116.9 (11.3)	105.9 (3.7)
Medical staffing vacancies, %^d^	13.1 (2.6)	14.2 (3.4)	13.1 (0.1)
Population			
Age ≥50 y, %	24.6 (0.1)	26.5 (1.2)	28.0 (0.3)
Race and ethnicity, %^e^			
Black	28.7	28.8	28.8
Hispanic	43.5	44.2	44.3
White	21.2	20.4	20.2
Other^f^	6.6	6.6	6.7
Chronic disease burden, %^g^			
High risk priority 1	5.7 (0.1)	6.5 (0.4)	7.0 (0.1)
High risk priority 2	8.7 (0.1)	9.2 (0.3)	9.5 (0.1)
Medium risk	34.1 (0.3)	33.9 (0.3)	35.3 (0.4)
Low risk	51.5 (0.1)	50.4 (1.0)	48.2 (0.3)
Patients with disability, %^h^	8.7 (0.2)	9.7 (0.5)	10.5 (0.1)

Abbreviation: CDCR, California Department of Corrections and Rehabilitation.

^a^ Data are means across the months in each year of the study period.

^b^ Data are from January 1 through July 31, 2021.

^c^ Defined as the ratio of a prison’s institutional population to its design capacity. Data on occupancy were missing for 2.9% of prison months.

^d^ Data on medical staffing vacancies were missing for 15.6% of prison months.

^e^ The CDCR provided only annual data on the race and ethnicity of the incarcerated population by prison as of June 30 of each year. Data were available for all but 1 facility.

^f^ Reported by the CDCR to include incarcerated people who were identified as American Indian, Filipino, and/or Asian and those whose race or ethnicity was unknown.

^g^ The CDCR defines low risk as patients with no chronic conditions or with well-controlled chronic conditions, medium risk as patients with 1 or more chronic illnesses, and high risk priority as patients with high-risk medical conditions, high health care costs and service use, and older age (the patients with the highest disease burden are placed in the priority 1 group).

^h^ Defined by the CDCR using Americans With Disabilities Act criteria.

**Figure.**
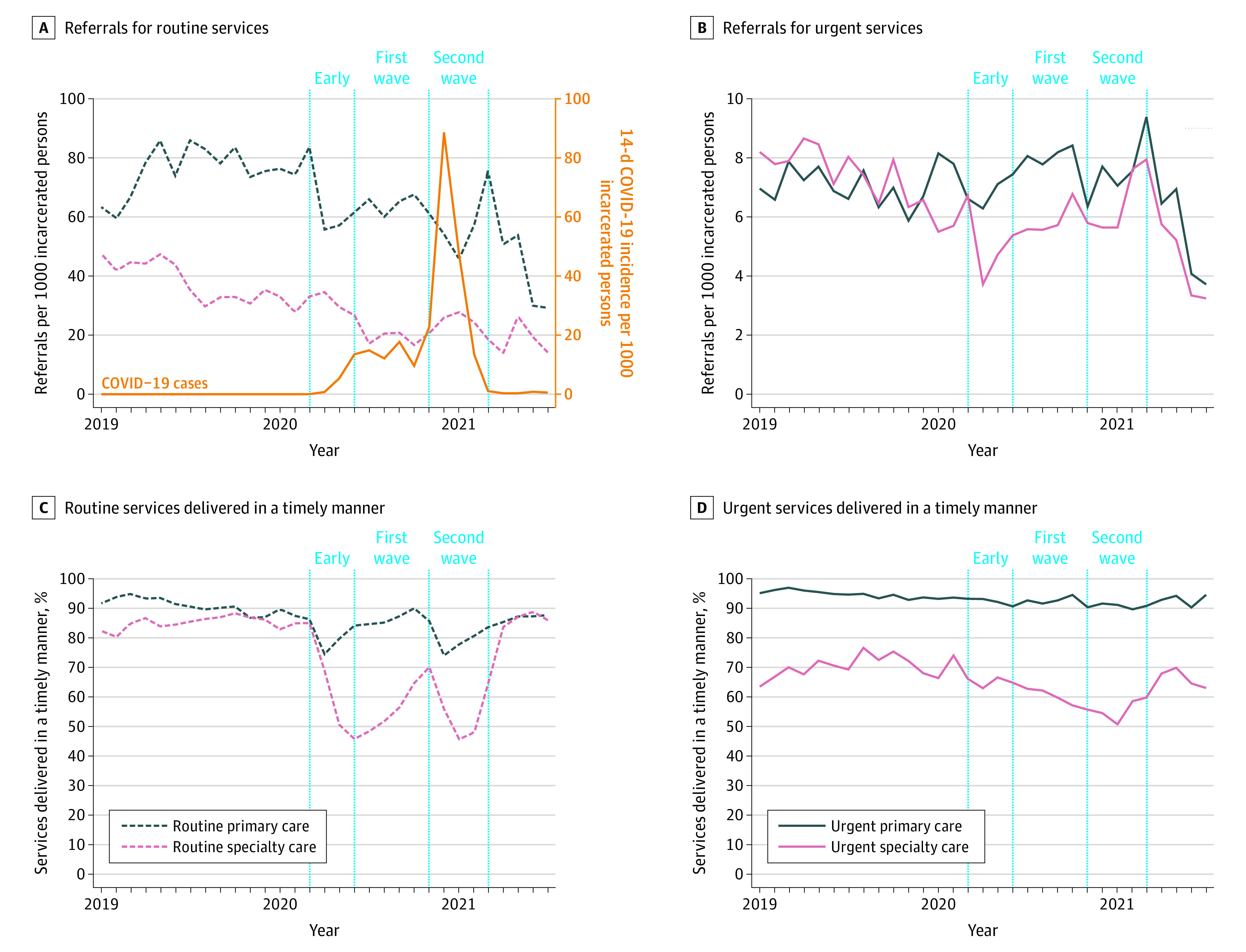
Delivery of Primary and Specialty Care to Incarcerated Persons in the California State Prison System Relative to COVID-19 Incidence From January 2019 to July 2021 All measures describe care delivered in person or through telemedicine. The eMethods in the Supplement gives definitions of measures, including health care types and timely delivery. Vertical dashed lines represent the early COVID-19 period (March to May 2020), first COVID-19 wave (June to October 2020), and second COVID-19 wave (November 2020 to February 2021); waves were defined as a mean 14-day COVID-19 incidence exceeding 10 cases per 1000 incarcerated persons for a given month. Data were missing for 2.7% of prison months for measures related to urgent primary care referrals.

Routine referrals decreased significantly during each COVID-19 wave compared with baseline for primary care (first wave: adjusted incidence rate ratio [aIRR], 0.74 [95% CI, 0.70-0.78]; second wave: aIRR, 0.63 [95% CI, 0.58-0.68]) and specialty care (first wave: aIRR, 0.58 [95% CI, 0.53-0.62]; second wave: aIRR, 0.67 [95% CI, 0.62-0.72]) (Figure, A). Although urgent specialty referrals decreased significantly during the first COVID-19 wave (aIRR, 0.73 [95% CI, 0.67-0.80]) and returned to baseline during the second wave, urgent primary care referrals remained stable during both waves (Figure, B).

The prison system experienced a significant decrease in the proportion of routine primary care delivered in a timely manner in the first wave (aIRR, 0.96 [95% CI, 0.93-0.99]) and second wave (aIRR, 0.90 [95% CI, 0.86-0.95]) but not in timeliness for urgent primary care (Figure, C and D). Timeliness for routine specialty care decreased significantly in the first wave (aIRR, 0.59 [95% CI, 0.56-0.63]) and second wave (aIRR, 0.64 [95% CI, 0.59-0.69]), whereas timeliness for urgent specialty care decreased significantly only in the second wave (aIRR, 0.77 [95% CI, 0.69-0.87]).

## Discussion

Although the California state prison population decreased during the COVID-19 pandemic, individuals who remained incarcerated experienced a higher burden of chronic disease and sustained, significant disruptions in referrals for and timely receipt of care. A limitation was that this study was restricted to a single state prison system and may not be representative of nationwide trends. Although the rate of specialty referrals was decreasing before the pandemic, all other measures had a stable baseline.

Given decreases in health care delivery during the pandemic, incarcerated persons may experience increased morbidity without concerted efforts to address care foregone during the pandemic. Policy makers should consider additional investments in prison medical resources to mitigate the impact of future pandemic waves.

## Notes

Supplement.
eMethods

eReferences
