# Supplementary material for: Access to Primary and Specialty Health Care in the California State Prison Population During the COVID-19 Pandemic
Source: JAMA Health Forum. 2022 Jul 15;3(7):e221868. doi: 10.1001/jamahealthforum.2022.1868 (PMC9287750; doi:10.1001/jamahealthforum.2022.1868)
Supplement: Supplement. — eMethods eReferences [file jamahealthforum-e221868-s001.pdf]

Supplementary Online Content

Amin A, Winetsky D, Schpero WL. Access to primary and specialty health care in the California state prison population during the COVID-19 pandemic. *JAMA Health Forum*. 2022;3(7):e221868. doi:10.1001/jamahealthforum.2022.1868

eMethods
eReferences

This supplementary material has been provided by the authors to give readers additional information about their work.

© 2022 Amin A et al. *JAMA Health Forum*.

eMethods

Data

Data on 14-day COVID-19 incidence for each prison from March 10, 2020 through July 31, 2021, were extracted from the California Department of Corrections and Rehabilitation (CDCR) COVID-19 prison tracking dataset.[1]

Information on receipt of primary and specialty care within the California state prison population came from the California Correctional Health Care Services (CCHCS) Dashboard, which was established in 2008 as a part of the federal receiver's "Turnaround Plan of Action" to improve medical care in California state prisons in response to *Brown v. Plata*.[2] This database includes monthly data for each prison between April 2012 and July 2021 on a variety of health care metrics, including population health management, access to care, staffing, costs, and demographics of the incarcerated population.[3] Our analysis focused on referrals for urgent and routine primary and specialty care services per 1,000 incarcerated persons, as well as the percent of services delivered in a timely manner. While the data on timeliness were publicly available, data on referral rates were obtained via Public Records Act request. Timeliness was defined by CCHCS as follows:

- **Urgent Primary Care Referrals**: Percentage of urgent primary care provider (PCP) referrals completed originating from a registered nurse (RN) face to-face triage appointment that are seen within one calendar day of referral.
- **Routine Primary Care Referrals**: Percentage of routine PCP referral orders completed originating from an RN face-to-face triage encounter that are seen within 14 calendar days of referral or as ordered, whichever occurs first.
- **Urgent (High Priority) Specialty Care Referrals**: The percentage of initial high priority specialty appointments that were completed within 14 calendar days of the referral or sooner as ordered by a PCP.
- **Routine Specialty Care Referrals**: The percentage of initial routine specialty appointments that occur per the provider's order and at least within 90 calendar days of the referral.

All variables describe in-person and telemedicine services requested or completed within a given month. CCHCS did not exclude care related to diagnosis and management of COVID-19 illness from these measures.

Prison characteristics included in the Table were defined by CCHCS as follows:

- **Institutional Population**: Institutional population on the last day of the reporting month.
- **Occupancy**: Percentage of the population design capacity occupied at each institution on the last day of the reporting month.
- **Medical Staffing Vacancies**: Monthly percentage of authorized positions within the Medical Services budget not filled by a civil service staff member.
- **Age ≥ 50**: Percentage of institutional population who are age 50 or older on the last day of the reporting month.
- **Race**: The CDCR Division of Correctional Policy Research and Internal Oversight provided race and ethnicity data by Public Records Act request from the Strategic

© 2022 Amin A et al. *JAMA Health Forum*.

Offender Management System, an internal electronic database that consolidates intake, history, and tracking of incarcerated persons and parolees. The CDCR reports that the “Other” category includes incarcerated people who have American Indian, Filipino, or Asian race in addition to those whose race or ethnicity is unknown.

- **Chronic Disease Burden**:
  - **High Risk Priority 1:** Percentage of patients who meet multiple criteria from a selection of evidence-based high-risk criteria which include sensitive medical conditions, multiple hospitalizations and/or emergency department visits, high risk specialty consultations, significant abnormal labs, high-risk diagnoses, high risk procedures, age, and high claims or pharmacy costs.
  - **High Risk Priority 2:** Percentage of patients who meet a single criterion from a selection of evidence-based high-risk criteria which include sensitive medical conditions, multiple hospitalizations and/or emergency department visits, high risk specialty consultations, significant abnormal labs, high-risk diagnoses, high risk procedures, age, and high claims or pharmacy costs.
  - **Medium Risk:** Percentage of patients with one or more chronic illnesses, based upon prescribed medications, laboratory test, or enrollment in the Mental Health Services Delivery System.
  - **Low Risk:** Percentage of patients with no chronic conditions other than well-controlled asthma, diabetes, hypertension, low risk hepatitis C viral infection, low risk latent tuberculosis infection; and who do not meet any of the criteria for High or Medium Risk.
- **Patients with Disability:** Percentage of institutional population who are ADA patients on the last day of the reporting month including those with vision, hearing, and mobility impairments. Excludes patients with developmental or learning disabilities.

Additional details on measure definitions can be found on the CCHCS Dashboard website.[4]

Statistical Analysis

We calculated changes in the delivery of care during the early COVID-19 period (March 2020-May 2020), first wave (June-October 2020), and second wave (November 2020-February 2021) compared to corresponding baseline levels during March 2019-May 2019, June-October 2019, and November 2019-February 2020, respectively. These changes were estimated using negative binomial models with prison fixed effects and time-varying controls for prison-level age distribution (proportion of incarcerated persons over age 50), chronic disease prevalence (proportion of incarcerated persons in High Risk Priority 1 category, as defined above), and disability prevalence (as defined above). For models examining changes in referrals, the offset was the number of incarcerated persons by prison month. For models examining the number of referrals delivered in a timely manner, the offset was the number of referrals by prison month. Unadjusted and adjusted results were qualitatively similar.

© 2022 Amin A et al. *JAMA Health Forum*.

eReferences

1. CDCR Population COVID-19 Tracking. California Open Data Portal. Accessed December 22, 2021. https://data.ca.gov/dataset/cdcr-population-covid-19-tracking

2. Kelso J. *Achieving a Constitutional Level of Medical Care in California's Prisons: The Federal Receiver's Turnaround Plan of Action*. California Prison Health Care Receivership Corp.; 2008. https://cchcs.ca.gov/wp-content/uploads/sites/60/2017/08/ReceiverTurnaroundPlan_060608.pdf

3. Reports & Court Orders. California Correctional Healthcare Services. Accessed December 22, 2021. https://cchcs.ca.gov/reports/#dashboard

4. Dashboard Glossary. CCHCS. Published 2020. https://cchcs.ca.gov/wp-content/uploads/sites/60/2018/02/Dashboard-Glossary.pdf

© 2022 Amin A et al. *JAMA Health Forum*.
